# The Incidence, Degree, and Timing of Hypocalcemia From Massive Transfusion: A Retrospective Review

**DOI:** 10.7759/cureus.22093

**Published:** 2022-02-10

**Authors:** Christopher P Potestio, Noud Van Helmond, Nadder Azzam, Ludmil V Mitrev, Akhil Patel, Talia Ben-Jacob

**Affiliations:** 1 Anesthesiology, Cooper Medical School of Rowan University, Camden, USA; 2 Anesthesiology, Cooper University Hospital, Camden, USA

**Keywords:** calcium repletion, hypocalcemia, massive transfusion protocol, massive transfusion, blood loss, hemorrhage

## Abstract

Background: Electrolyte administration during massive transfusion without readily available calcium laboratory values is likely ubiquitous but not well standardized. We aimed to quantify the incidence, degree, and timing of hypocalcemia during the first 24 hours after initiation of a massive transfusion with the institutional massive transfusion protocol (MTP). We hypothesized that hypocalcemia is prevalent during acute resuscitation (first six hours) despite efforts of the treatment team to replete calcium during active resuscitation.

Methods: A retrospective chart review of all patients who underwent MTP at our institution between January 1, 2017, and December 31, 2017, was performed. The primary outcome was hypocalcemia from a massive transfusion during the first six hours after the initiation of the MTP. Secondary outcomes of interest included hypercalcemia, hypomagnesemia, hospital mortality, peak and nadir timing of hypocalcemia and hypercalcemia, calcium supplementation, and calcium supplementation timing. Calcium administration and blood product transfusion is reported relative to the start of the MTP. The association between the total amount of calcium administered and the total number of blood products transfused was assessed.

Results: Data from 52 massive transfusions were analyzed. Ninety-seven percent of patients were hypocalcemic during the first six hours of resuscitation. The nadir occurred after median of eight units of blood product were given, (interquartile range {IQR}: 4-16). Calcium supplementation correlated with the total number of blood products transfused (ρ = 0.47, p < 0.01). Patients in whom calcium was supplemented received more blood products when compared to patients in whom calcium was not supplemented (median: 16, IQR: 12-26 vs. median: 9, IQR: 7-12, p <0.01).

Conclusions: Hypocalcemia from massive transfusion is common. The incidence of hypocalcemia in MTP has been reported to be 85-97%. Calcium supplementation that is not standardized in MTP may lead to underutilization during massive transfusion and to hypocalcemia in these patients.

## Introduction

Massive transfusion is often necessary for the treatment of acute hemorrhagic shock. However, transfusion of large volumes of blood products is not a benign therapy. Massive transfusion replaces hemorrhaged whole blood with component blood products, leading to dilutional coagulopathy [1]. Massive transfusion is also associated with hypocalcemia due to citrate toxicity, among other electrolyte abnormalities [2]. Other complications include hypothermia if transfused without a warmer, which in the setting of trauma can lead to further deterioration of the patient [1].

Many institutions have standardized massive transfusion protocols (MTP) to promote efficient acute resuscitation while minimizing the side effects of massive resuscitation [3]. In addition to the standardized replacement of intravascular volume, MTPs typically aim to correct trauma-induced coagulopathy to curb further blood loss. Optimizing the ratio of units of packed red blood cells (PRBCs) to units of plasma may help prevent dilutional coagulopathy [3]. There are no published guidelines addressing the management of hypocalcemia, even though citrate toxicity is a well-established side effect of massive transfusion and hypocalcemia can worsen coagulopathy [4].

Citrate toxicity in massive transfusion occurs when citrate administration exceeds citrate metabolism. It takes a non-cirrhotic adult five minutes to metabolize 3 g of citrate found in one unit of packed red blood cells [5]. Metabolism of citrate can take even longer in patients with liver dysfunction. Citrate binds to calcium, leading to rapid hypocalcemia during massive transfusion. Hypocalcemia leads to hypotension and coagulopathy, both of which can be devastating during massive transfusion.

Current recommendations are based on information extrapolated from animal studies and involve checking electrolyte levels frequently during massive transfusion, but during acute resuscitation in centers where point of care testing is not available, waiting for the laboratory chemistry results is not a pragmatic option [6]. PRBCs in the United States are stored in solutions containing as much as 3 g of citrate per unit of blood, with an even greater concentration of citrate in plasma and platelets [4]. As resuscitation efforts in MTPs shift toward citrate-rich plasma, where a 1:1 ratio of PRBC to plasma replaces a 1:2 ratio, addressing hypocalcemia as a side effect of massive resuscitation will become increasingly important.

In a single-center retrospective review of trauma patients who received massive transfusion following a 1:1 PRBC to plasma ratio, 97% (n = 152) of patients were noted to have hypocalcemia; 71% of these patients were noted to have severe hypocalcemia (ionized calcium <3.6 mg/dL) [7]. Patients with severe hypocalcemia had higher lactate, lower pH, and higher mortality. While calcium is important in coagulation, it also plays a critical role in neuromuscular and cardiovascular membrane stability, myocardial contractility, and cardiac conduction. Clinical signs of hypocalcemia include heart failure, prolonged QT interval, sinus tachycardia, paresthesia, muscle spasms, cramps, tetany, circumoral numbness, and seizures [8]. Given the integral role that calcium plays in coagulation and hemodynamics, it is no surprise that several studies have shown a correlation between hypocalcemia and mortality [7,9-11].

Calcium administration during a massive transfusion without readily available calcium laboratory values is likely ubiquitous but not well standardized in MTPs. Therefore, observational data on calcium concentrations during massive transfusion may be of value to establish estimated calcium concentration trends and time intervals for calcium supplementation.

Our primary aim was to quantify the incidence, degree, and timing of hypocalcemia during the first 24 hours after initiation of a massive transfusion with the institutional MTP. A secondary aim was to explore calcium supplementation patterns during massive transfusion. We hypothesized that hypocalcemia is prevalent during acute resuscitation despite efforts of the treatment team to replete calcium during active resuscitation.

## Materials and methods

We performed a consecutive retrospective chart review on patients for whom the institutional MTP was initiated. In adults, several definitions of massive transfusion exist based on the volume of the blood products transfused, with the three most common definitions of MTP being (1) transfusion of ≥10 units within 24 hours, (2) transfusion of >4 units in one hour with anticipation of continued need for blood product support, and (3) replacement of >50% of the total blood volume by blood products within three hours [12]. In line with these definitions, we included patients who received more than four units after the initiation of the MTP or patients who received more than 10 units of blood product over 24 hours after MTP was initiated. Patients were included from January 1, 2017, to December 31, 2017, in a 600-bed level one trauma center in a highly populated, the United States East Coast, urban setting. Our institutional review board approved the study (Cooper University Health Care Institutional Review Board; approval number: 18-115) and waived the requirement to obtain informed consent.

Massive transfusion protocol

Blood products included PRBCs, whole blood, thawed plasma, platelets, or cryoprecipitate. The MTP at our institution follows a 1:1 ratio of PRBCs to plasma, with one pack of pooled platelets administered for every four units of PRBCs/plasma. MTP packs are supplied at bedside immediately upon request. Each MTP pack contains three units of O+ PRBCs and three units of A plasma for males and three units of O- PRBCs and three units of A plasma for females. Two units of single donor platelets are available at all times during the MTP. At least five MTP packs are maintained within the blood bank at all times.

Electrolytes are replaced at the discretion of the clinician. Blood products at our institution are suspended in one of three adenine-saline solutions (AS-1, AS-3, AS-5) which contain adenine, saline, glucose, citrate, and phosphate [13]. The amount of citrate in these solutions is approximately 0.042 g/100 mL. Each unit of blood contains between 320 mL and 360 mL of volume.

Calcium chloride was used for all calcium repletion. Calcium chloride is preferable to calcium gluconate because the solute in calcium chloride is readily available upon injection into the bloodstream. Calcium gluconate, on the other hand, requires adequate liver blood flow and liver function in order to metabolize gluconate and release elemental calcium. Patients with acute hemorrhage experience decreased liver blood flow and impaired liver function and calcium gluconate may be ineffective [6].

Clinical data collection

Demographic and clinical information was retrieved from the electronic medical record (Epic; Verona, WI: Epic Systems). Data collected included age, duration of massive transfusion, indication for transfusion, units of blood product administered (including administration of whole blood), dose of calcium chloride administered, arterial blood gas analyses, blood counts, ionized calcium, platelet count, and chemistry panel including total serum calcium. Time from the start of the massive transfusion was also recorded for each laboratory value. The start time of the massive transfusion was defined as the time the blood bank received the call to issue blood products in the context of the MTP. All laboratory, clinical, and treatment data for the first 24 hours after initiation of the MTP were collected. We recorded the need for cardiopulmonary resuscitation, need for dialysis, and the need for other procoagulant therapies such as tranexamic acid and concentrated clotting factors.

Outcomes

The primary outcome was hypocalcemia from massive transfusion during the first six hours after the initiation of the MTP. Hypocalcemia was defined as a total serum calcium level below 8.5 mg/dL or whole blood ionized calcium below 4.5 mg/dL. These are the lower limits of normal (8.5-10.5 mg/dL total serum calcium and 4.5-5.24 mg/dL whole blood ionized calcium) established by our chemistry laboratory. Severe hypocalcemia was defined as total serum calcium level below 6.5 mg/dL, or ionized calcium below 2.0 mg/dL, based on previous studies on hypocalcemia during massive transfusion [14]. Both serum calcium level and ionized calcium level were used in this analysis because the former is measured in the intensive care unit while the latter is used in the acute setting of an MTP where it is impracticable to wait upwards of an hour for a result. Secondary outcomes of interest included hypercalcemia, hospital mortality, peak and nadir timing of hypocalcemia and hypercalcemia, calcium supplementation, and calcium supplementation timing. Hypercalcemia was defined as total serum calcium level above 10.5 mg/dL or whole blood ionized calcium above 5.24 mg/dL.

Data and statistical analysis

We excluded data from analysis if blood product transfusion times were not accurately registered in the electronic medical records (EMR) (e.g., 40 units administered at a single time point). The Shapiro-Wilk test was used to assess the distributions of the data. Group data for continuous variables are presented as mean with standard deviation (SD) or as median with interquartile range (IQR), depending on the distribution of the data. Categorical data are presented as n (%). For visual presentation of the data, calcium concentrations were normalized as a percentage of the lower limit of the normal concentration range to allow for integrated presentation of whole blood ionized calcium values as well as total serum calcium concentrations. Median nadir and peak calcium concentrations and their timing were calculated and graphed. We plotted when calcium supplementation was given in relation to the number of blood products transfused and we assessed the association between the total amount of calcium administered versus the total number of blood products transfused using Spearman rank-order correlation. Normally distributed data were compared using unpaired t-test while the Mann-Whitney rank-sum test was used for non-normally distributed data. P-values are reported for statistical tests.

## Results

We included 51 adult patients who underwent massive transfusion using MTP in the study period. One patient received MTP on two separate occasions during hospitalization, these were counted as individual encounters, as there were more than 24 hours passed between events. Patients in whom MTP was initiated but who eventually received less than four units were excluded from the study. Thus, data from 52 massive transfusions encounters was analyzed. Patient demographics and clinical characteristics are presented in Table 1. Laboratory values in Table 1 represent the first registered values after the initiation of the MTP.

**Table 1:** Baseline demographics and clinical characteristics. *Lab values represent the first recorded value after the initiation of the massive transfusion protocol. **Estimated blood loss was extracted from surgical notes and the recorded value was sometimes much lower than the total blood loss that might have happened before arrival.

Demographics and presentation	
Age in years, mean ± SD (range)	52 ± 20 (18-89)
BMI in kg/m^2^, mean ± SD (range)	29 ± 9 (17-60)
Sex, n (%) male / n (%) female	36/16 / (69/31)
Type of inciting event, n (%)	
Traumatic bleeding	25 (48)
Non-traumatic bleeding	27 (52)
pH, mean ± SD (range)*	7.16 ± 0.16 (6.81-7.46)
Total serum calcium in mg/dL, mean ± SD (range)*	8.5 ± 2.1 (5.7-16.8)
Ionized calcium in whole blood in mg/dL, mean ± SD (range)*	3.5 ± 1.0 (2.0-5.8)
Serum potassium, mean ± SD (range)*	4.3 ± 0.9 (2.7-7.7)
Hemoglobin in g/dL, mean ± SD (range)*	9.1 ± 2.5 (5.1-15.7)
Platelets 10^9 ^× L^-1^, mean ± SD (range)*	114 ± 67 (9-268)
International normalized ratio, mean ± SD (range)*	1.5 ± 0.4 (1-2.8)
Mg, mean ± SD (range)*	1.8 ± 0.4 (1.1-2.8)
Massive transfusion characteristics	
Units of packed red blood cells administered, mean n ±SD (range)	13 ± 10 (3-50)
Units of thawed plasma administered, mean n ± SD (range)	12 ± 9 (0-40)
Units of pooled platelets administered, mean n ± SD (range)	2 ± 2 (0-9)
Units of total blood product administered, mean n ± SD (range)	27 ± 22 (5-97)
Units of whole blood administered, mean n ± SD (range)	0 ± 1 (0-2)
Total calcium supplementation administered in g, mean ± SD (range)	3.5 ± 2.9 (0-10)
Massive transfusion in which tranexamic acid administered, n (%)	12 (23)
Estimated blood loss in mL, mean ± SD (range)	2895 ± 3210 (10-16000)**
Requiring cardiopulmonary resuscitation during massive transfusion, n (%)	22 (42)
In hospital follow-up	
Post-resuscitation shock, n (%)	28 (54)
Requiring hemodialysis during hospitalization, n (%)	6 (12)
Venous thromboembolism during hospitalization, n (%)	9 (17)
Survival to hospital discharge, n (%)	19 (37)

Incidence of hypocalcemia

**Figure 1:**
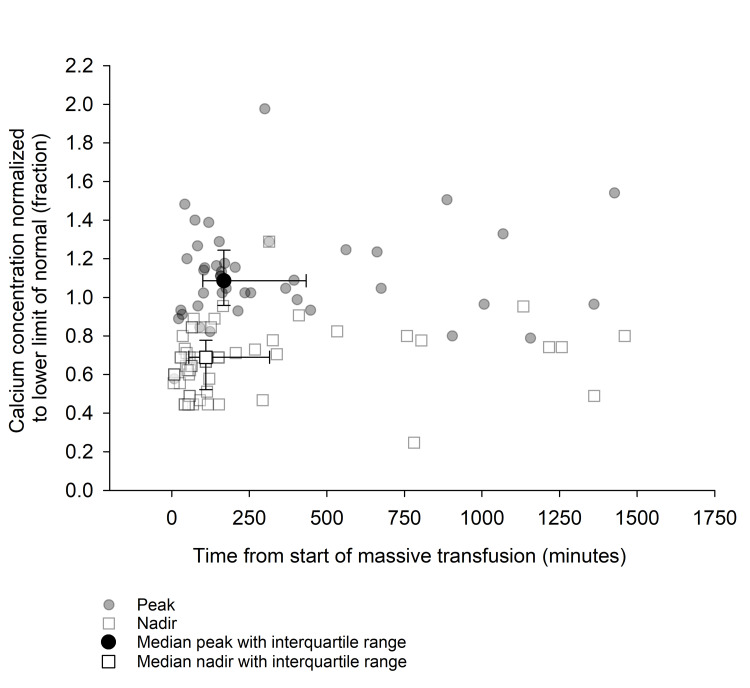
Individual and median calcium nadir and peak values.

During the 52 massive transfusions, 50 (97%) patients were hypocalcemic and 42 (81%) patients were severely hypocalcemic during the first six hours of resuscitation. Figure 1 shows individual and median calcium nadir and peak values from the beginning of the massive transfusion normalized to the lower limits of ionized calcium and serum calcium. The median calcium nadir relative to the lower limits of normal occurred at 110 minutes (IQR: 55-315) from the start of massive transfusion. The absolute median nadir whole blood ionized calcium was 2.9 mg/dL (IQR: 2.2-3.3). The absolute median nadir total serum calcium was 6.3 (IQR: 6.1-7.4 mg/dL). The nadir occurred after a median of eight units (IQR: 4-16) of blood products were given. For 13 (25%) of 52 massive transfusions, there was no calcium concentration measurement in the normal range within 24 hours after the initiation of the MTP. Only one patient (3%) was hypercalcemic during the first 24 hours after the MTP was initiated. The median calcium peak relative to the lower limits of normal occurred at 168 minutes (IQR: 100-433) from the start of massive transfusion and after a median total of 12 (IQR: 6-19) blood products were transfused. The median absolute peak whole blood ionized calcium was 4.9 mg/dL (IQR: 4.1-5.4). The median absolute peak total serum calcium was 9.2 mg/dL (IQR: 8.6-11.0).

Calcium supplementation during massive transfusion

**Figure 2:**
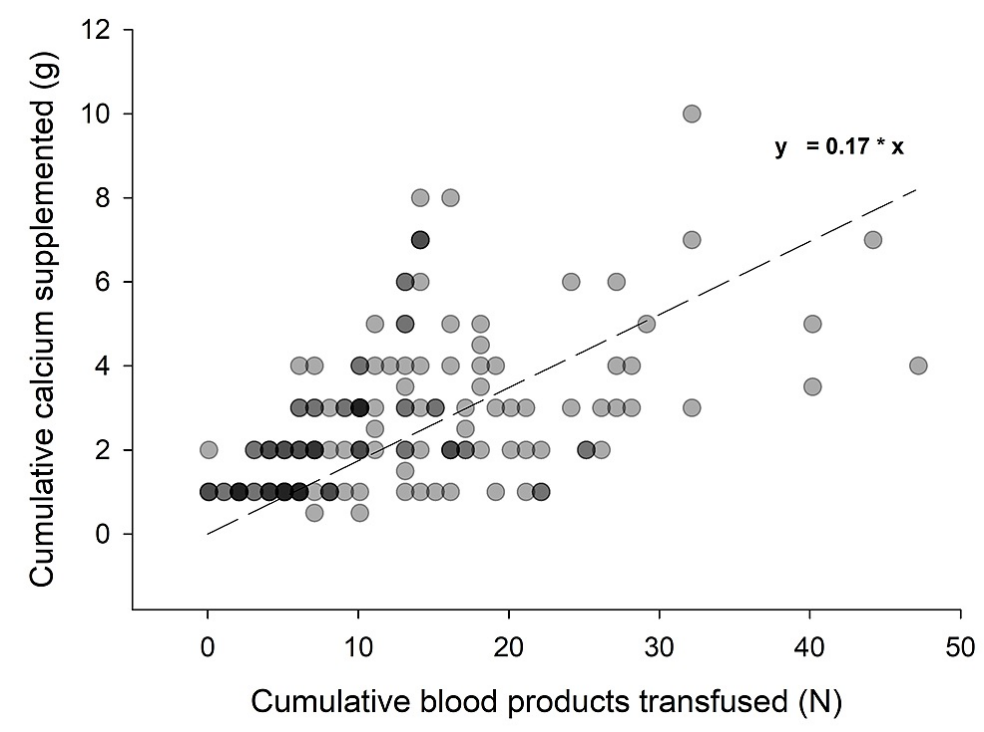
Cumulative calcium supplementation.

Cumulative calcium supplementation for each individual patient who received supplementation (n = 44, 85%) is depicted in Figure 2. In these patients, 0.17 g of calcium chloride was given for each blood product transfused. Incremental calcium supplementation appeared to be the best fitted with a straight line, indicating that supplementation was likely similar early in massive transfusions versus later in the massive transfusions. Patients in whom calcium was supplemented received more blood products when compared to patients in whom calcium was not supplemented (median: 16, IQR: 12-26 vs. median: 9, IQR: 7-12, p < 0.01).

## Discussion

Principal findings

This study aimed to quantify the incidence, degree, and timing of hypocalcemia during the first 24 hours after initiation of a massive transfusion using MTP. To our knowledge, this is the only study to address the time course of hypocalcemia during MTP. Consistent with our hypothesis, we found that 97% of patients were hypocalcemic during acute resuscitation (first six hours) despite efforts of the treatment team to replete calcium during the resuscitation. The nadir of calcium values tended to be earlier during massive transfusion than the peak of calcium values. Median peak values of whole blood ionized calcium and total serum calcium were just above the lower limits of the normal range. For a substantial proportion of patients, no normocalcemic values were registered within 24 hours after the initiation of the MTP.

Previous studies on hypocalcemia from massive transfusion

Giancarelli et al. conducted a retrospective review of trauma patients who received massive transfusion in a single center in the southern United States [7]. Similar to our results, most patients suffered severe hypocalcemia (71%), which was associated with higher mortality. Our results showed a higher percentage of patients with severe hypocalcemia which may be due to the fact that we presented all patients who underwent MTP and not just trauma patients. Given that some of our MTP patients may be transferred to non-surgical services who may not be as aware of the side effect of hypocalcemia, there could potentially be delayed responses in laboratory assessments and supplementation. Giancarelli et al. only looked at ionized calcium at one moment in time whereas we looked over a period to review a trajectory and assess for potential timing of intervention. Several recent studies have measured the incidence of hypocalcemia during massive transfusion and found similarly high incidences [7,9-11].

In addition, other studies have examined calcium levels in less acute settings such as the mixed ICU population [15] and trauma ICU patients receiving total parenteral nutrition [16,17]. Incidence of hypocalcemia has been reported to be 55% in these patients, but the calcium levels in those studies may not apply to the acute resuscitation of an MTP where the rate of blood product transfusion exceeds the rate of citrate metabolism. In addition, the physiologic effects of hypocalcemia - decreased myocardial contractility and impaired coagulation - are detrimental to the bleeding patient and may be better tolerated by other ICU populations.

Mechanisms underlying observations

It takes a healthy liver five minutes to metabolize 3 g of citrate in a single unit of packed red blood cells, thus, transfusion of blood products faster than one unit every five minutes will reliably and predictably lead to citrate toxicity. In addition, patients experiencing acute blood loss are likely to suffer hepatic injury and impaired hepatic function as a result of variable hepatic blood flow. Transfusion rates greater than one unit of PRBCs per five minutes and liver dysfunction are both common during MTP, and both lead to citrate level elevation and hypocalcemia [4,14,18]. The high rate of transfusion in MTP leads to high risk of severe hypocalcemia, which explains the high incidence of severe hypocalcemia in this study (82% in the first six hours, 85% in 24 hours). 

Our findings confirm a dose-response relationship between the volume of blood products administered and calcium supplementation administered, yet many patients remained hypocalcemic. Recommendations extrapolated from animal studies have suggested using mathematical equations to quantify the amount of calcium needed given rate of transfusion and citrate load but no formal guidelines have been published nor has this method been proven effective. Further studies need to be performed in humans to determine the correct dose-response relationship.

Clinical implications

The inciting event that causes a provider to activate MTP is usually acute bleeding and the majority of transfusion happens during the first 120 minutes of transfusion. The rapid rate of transfusion likely exceeds the liver’s capacity to metabolize citrate, predictably leading to severe hypocalcemia. Severe hypocalcemia is most often seen during the first 120 minutes after initiation of MTP, as evidenced by the nadir calcium levels in Figure 2. The peak calcium levels occur more than 240 minutes after initiation of MTP. Incidence of hypocalcemia in MTP has been reported to be 85-97% when similar definitions of hypocalcemia are used [7,10]. With more aggressive calcium repletion early in the resuscitation during MTP, providers can avoid the predictable severe hypocalcemia associated with rapid transfusion.

Calcium is required by several clotting factors for activation, II, VII, IX and X, and proteins C and S. In addition, calcium plays a role in stabilizing fibrinogen and platelets in the developing thrombus [7,19]. Contractility of myocardial and smooth muscle cells is dependent on calcium. Hypocalcemia can lead to coagulopathy, myocardial depression, and vasodilation: physiologic changes that complicate the management of hemorrhagic shock.

Only one of our patients (3%) was hypercalcemic at any point during the first 24 hours after initiation of MTP and that patient’s serum calcium was only slightly elevated at 11.2, a value that is above the normal range but not high enough to cause arrhythmia, muscle weakness, or other negative side effects of hypercalcemia. Our data suggest that there is clear room for improvement in calcium repletion during MTP. Even though hypocalcemia is a predictable side effect of rapid transfusion, calcium repletion at our institution was not rapid or aggressive enough to maintain normal calcium levels during the first 120 minutes after initiation of MTP.

Our providers administered 0.17 g of calcium chloride per blood product administered during MTP. This rate of administration led a majority of patients to suffer from hypocalcemia with a very low incidence of hypercalcemia. Other studies have recommended between 0.5 g and 1.0 g of calcium chloride per blood product administered [6,7]. Our data suggest that, despite knowledge of citrate toxicity, providers commonly underestimate the amount of calcium needed during MTP and rarely, if ever, overestimate. Future studies are needed to determine whether such a recommendation will lead to additional calcium supplementation and less incidence of hypocalcemia.

Our study has several limitations related to the retrospective design. EMR registration may not accurately reflect the timing of transfusion or supplementation. Due to the acute nature of resuscitation during MTP, calcium supplementation may be charted in retrospect which may lead to inaccurate recording and possibly recall bias. Both supplementation of calcium and more frequent lab measurements may be triggered by more severe bleeding. Findings may therefore not relate to all massive transfusions. There was also substantial variability between patients that may make it challenging to incorporate calcium supplementation in a standardized fashion (i.e., “g calcium/blood product”). 

It is not obligatory to measure serum calcium levels prior to the administration of calcium. It is unclear whether calcium supplementation described in this study is empiric therapy or the result of measured serum calcium levels. Future studies may investigate the amount of calcium given empirically versus the amount given as the result of measured serum calcium levels.

We did not address the dose-response relationship of calcium dosing during MTP nor has this been addressed in any previous study. It would be ideal to be able to standardize calcium administration, but no study has been able to establish this relationship. One crucial variable that affects the administration of calcium during MTP is the speed of transfusion. A slower transfusion of blood, e.g., one unit every 10 minutes will require less calcium dosing than a rapid transfusion speed of one unit every 30 seconds via a rapid transfusion device. The variability of speed of transfusion between patients makes calcium dosing a dynamic target. MTP activation will usually result in high-speed transfusion that will overcome the speed of citrate removal by the liver, hence necessitating calcium administration as seen by our study.

It is clear that the provider should target normal calcium levels in patients undergoing rapid transfusion. However, it is unclear whether more aggressive calcium supplementation would have a meaningful impact on patient outcomes. Further studies should aim to establish this relationship.

## Conclusions

Hypocalcemia from massive transfusion is common. Calcium supplementation was positively correlated with the total number of blood products transfused and yet hypocalcemia remained prevalent. Lack of standardization of calcium supplementation in MTP is likely leading to underutilization of calcium during massive transfusion. Despite recognition of citrate toxicity as a common side effect of MTP, calcium repletion is insufficient to avoid hypocalcemia, especially during the first hours of resuscitation.

## References

[1] Levy JH (2006). Massive transfusion coagulopathy. Semin Hematol.

[2] Dzik WH, Kirkley SA (1988). Citrate toxicity during massive blood transfusion. Transfus Med Rev.

[3] Riskin DJ, Tsai TC, Riskin L (2009). Massive transfusion protocols: the role of aggressive resuscitation versus product ratio in mortality reduction. J Am Coll Surg.

[4] Li K, Xu Y (2015). Citrate metabolism in blood transfusions and its relationship due to metabolic alkalosis and respiratory acidosis. Int J Clin Exp Med.

[5] Kramer L, Bauer E, Joukhadar C, Strobl W, Gendo A, Madl C, Gangl A (2003). Citrate pharmacokinetics and metabolism in cirrhotic and noncirrhotic critically ill patients. Crit Care Med.

[6] Stainsby D, MacLennan S, Thomas D, Isaac J, Hamilton PJ (2006). Guidelines on the management of massive blood loss. Br J Haematol.

[7] Giancarelli A, Birrer KL, Alban RF, Hobbs BP, Liu-DeRyke X (2016). Hypocalcemia in trauma patients receiving massive transfusion. J Surg Res.

[8] Fong J, Khan A (2012). Hypocalcemia: updates in diagnosis and management for primary care. Can Fam Physician.

[9] Hall C, Nagengast AK, Knapp C (2021). Massive transfusions and severe hypocalcemia: an opportunity for monitoring and supplementation guidelines. Transfusion.

[10] MacKay EJ, Stubna MD, Holena DN (2017). Abnormal calcium levels during trauma resuscitation are associated with increased mortality, increased blood product use, and greater hospital resource consumption: a pilot investigation. Anesth Analg.

[11] Chanthima P, Yuwapattanawong K, Thamjamrassri T (2021). Association between ionized calcium concentrations during hemostatic transfusion and calcium treatment with mortality in major trauma. Anesth Analg.

[12] Pham HP, Shaz BH (2013). Update on massive transfusion. Br J Anaesth.

[13] D'Amici GM, Mirasole C, D'Alessandro A, Yoshida T, Dumont LJ, Zolla L (2012). Red blood cell storage in SAGM and AS3: a comparison through the membrane two-dimensional electrophoresis proteome. Blood Transfus.

[14] Lim F, Chen LL, Borski D (2017). Managing hypocalcemia in massive blood transfusion. Nursing.

[15] Steele T, Kolamunnage-Dona R, Downey C, Toh CH, Welters I (2013). Assessment and clinical course of hypocalcemia in critical illness. Crit Care.

[16] Dickerson RN, Morgan LG, Cauthen AD, Alexander KH, Croce MA, Minard G, Brown RO (2005). Treatment of acute hypocalcemia in critically ill multiple-trauma patients. JPEN J Parenter Enteral Nutr.

[17] Dickerson RN, Morgan LM, Croce MA, Minard G, Brown RO (2007). Treatment of moderate to severe acute hypocalcemia in critically ill trauma patients. JPEN J Parenter Enteral Nutr.

[18] Uhl L, Maillet S, King S, Kruskall MS (1997). Unexpected citrate toxicity and severe hypocalcemia during apheresis. Transfusion.

[19] Elmer J, Wilcox SR, Raja AS (2013). Massive transfusion in traumatic shock. J Emerg Med.

